# A Tribute to the Legendary Physician and Politician: Dr. Bidhan Chandra Roy

**DOI:** 10.7759/cureus.73222

**Published:** 2024-11-07

**Authors:** S Johnson, Helan Rajan, Sheeza Shaikh, Deepu Palal, Prerna Verma, Nidhi Shree

**Affiliations:** 1 Community Medicine, Dr. D. Y. Patil Medical College, Hospital and Research Centre, Dr. D. Y. Patil Vidyapeeth (Deemed to be University), Pune, IND; 2 Otolaryngology, Dr. D. Y. Patil Medical College, Hospital and Research Centre, Dr. D. Y. Patil Vidyapeeth (Deemed to be University), Pune, IND

**Keywords:** dr. bidhan chandra roy, historical vignettes, indian healthcare reform, medical council of india (mci), physician writer, politician, public health education

## Abstract

This comprehensive review article examines the profound and multifaceted impact of Dr. Bidhan Chandra Roy (1 July 1882 to 1 July 1962) on the political landscape and healthcare system of post-independence India, with a particular focus on West Bengal. Dr. Roy, a distinguished physician who became the second Chief Minister of West Bengal, embodied a rare combination of medical expertise and political acumen that significantly shaped the trajectory of the newly independent nation. The article delves into Dr. Roy's pivotal role in West Bengal's industrialisation, including establishing the Durgapur Steel Plant and the Kalyani engineering hub, which laid the foundation for the state's economic development. It also explores his visionary urban planning initiatives, exemplified by the creation of Salt Lake City (Bidhannagar), which revolutionised Kolkata's expansion. The review analyses Dr. Roy's transformative contributions to healthcare, including establishing numerous medical institutions such as the Chittaranjan Cancer Hospital and the Chittaranjan Seva Sadan Hospital for women and children. His efforts to improve public health infrastructure, enhance medical education, and raise the overall standard of healthcare delivery in India are critically examined. The article investigates how Dr. Roy's medical background informed his political decisions, leading to innovative, evidence-based approaches to addressing public health challenges and implementing welfare schemes. His instrumental role in founding the Indian Medical Association and his influence on shaping national health policies are also scrutinised. This review provides a holistic understanding of Dr. Roy's enduring legacy by synthesising historical records, policy documents, personal accounts, and contemporary analyses. It argues that his integrated approach to governance and medicine transformed West Bengal and set a precedent for evidence-based policymaking in India, particularly in the health sector. The article concludes by reflecting on how Dr. Roy's vision and principles continue to be relevant in addressing contemporary challenges in public health and governance, cementing his status as a visionary leader whose impact transcends regional and national boundaries.

## Introduction and background

**Figure 1:**
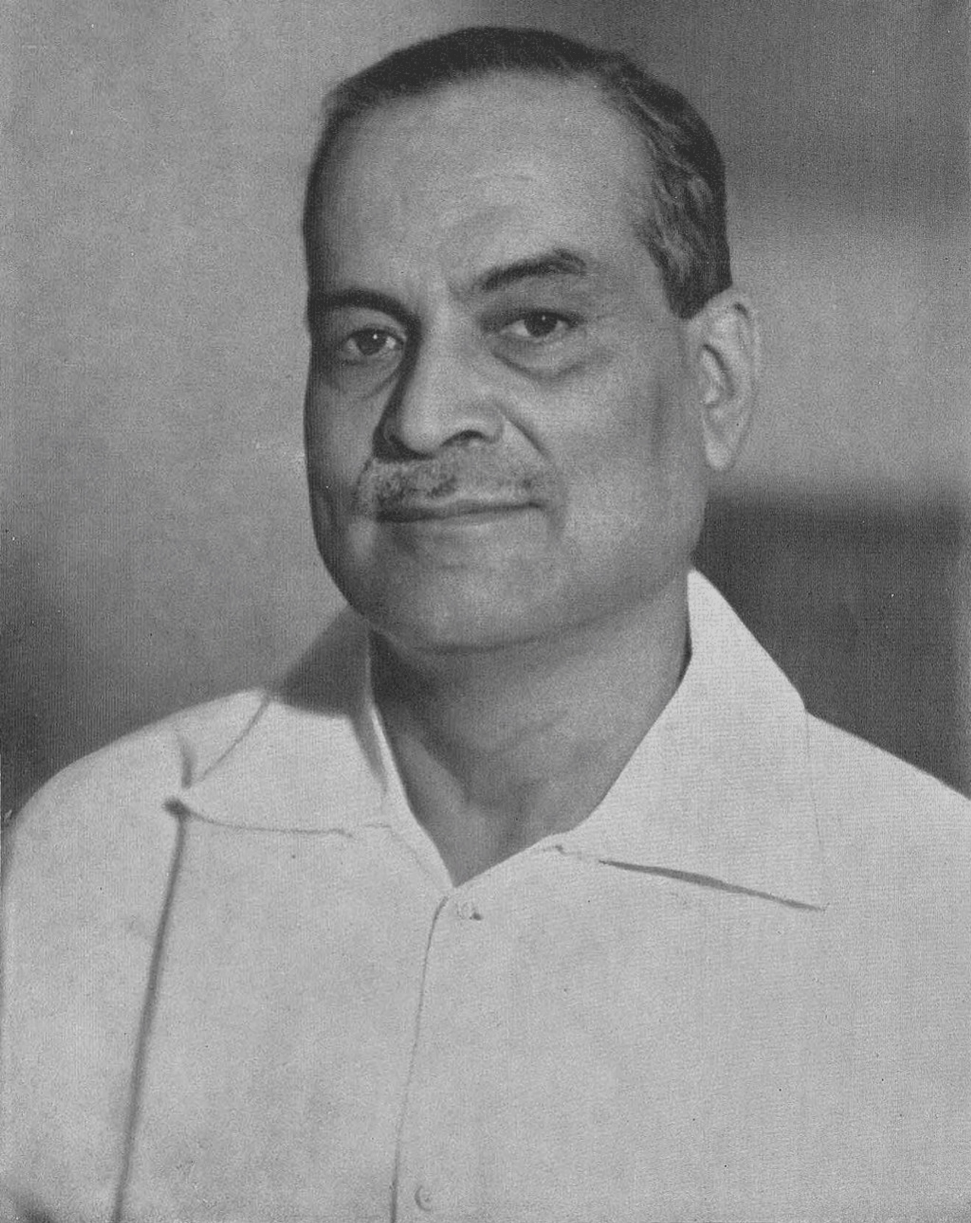
Dr. Bidhan Chandra Roy Source: [5]

Dr. Bidhan Chandra Roy (1 July 1882 to 1 July 1962) was an eminent Indian physician and politician (Figure 1). He was born on 1 July 1882, in Bankipore, Patna, to Prakash Chandra Roy, an excise inspector, and Aghore Kamini Devi. At that time, Patna was included within the boundaries of United Bengal, and this period, known as the British Raj, lasted from 1858 to 1947. During this time, many Indians were pushing for freedom from British rule. Dr. Roy grew up, and the movement for India's independence was gaining strength. Leaders such as Mahatma Gandhi were using non-violent methods to challenge British authority. Dr. Roy's adult life coincided with World War I (1914-1918) and World War II (1939-1945). He was pivotal in advancing various cities, including Salt Lake, now part of the Bidhannagar Municipal Corporation, Kalyani, and Durgapur. In recognition of his invaluable contributions, National Doctor's Day is observed in India every year on 1 July. In 1961, Dr. Roy was awarded the Bharat Ratna, India’s highest civilian honour, for his exceptional service to the nation [1]. The first National Doctors' Day was celebrated on 1 July 1991, in recognition of Dr. Roy, popularly known as B.C. Roy, who was the second Chief Minister of West Bengal. This significant date commemorates his birth and death anniversaries [2]. Today, medical professionals across the Indian subcontinent are celebrating the anniversary of Dr. Roy, recognising him as one of the most eminent physicians in contemporary history [3]. Dr. Roy's experience as a physician profoundly influenced his political career, mainly his focus on public health and the development of medical infrastructure in India. Dr. Roy asserted that attaining India's freedom is contingent upon physically and mentally healthy individuals [4]. Dr. Roy lived through significant periods of Indian history, including British colonial rule and India’s early years of independence. Dr. Roy’s pioneering work in healthcare infrastructure and medical education provides valuable insights into the global development of modern healthcare systems and, undoubtedly, into Indian politics. Dr. Roy's life and work provide valuable insights into how countries such as India navigated the transition from colonial rule to independence, balancing traditions with modernisation. His efforts to improve healthcare and education while engaging in politics and urban planning demonstrate the multifaceted approach many leaders took in newly independent nations during the mid-20th century. By studying Dr. Roy's contributions, we can better understand the challenges and opportunities many countries faced as they developed modern healthcare systems and political structures in the decades following World War II. His biography is essential for India and offers lessons and perspectives relevant to global history and development.

## Review

Dr. Roy’s life and career

The Patna district of Bihar is home to Dr. Roy's birth and upbringing. His mother was a dedicated social worker, and his father worked as an excise inspector. His mother taught him the Bhagavad Gita and the writings of Rabindranath Tagore, which greatly influenced his personality. Dr. Roy graduated with a Bachelor of Arts degree, achieving honours in Mathematics from Patna College. In 1901, he was accepted into both the Indian Institute of Engineering Science and Technology and Calcutta Medical College, but he chose to enrol in Calcutta Medical College. His time there was marked by significant hardships. As his father was no longer in the workforce, he needed to earn a living to support himself. He was able to secure scholarships to help alleviate the financial burden of his education [6].

The words ‘Whatever thy hands findeth to do, do it with thy might’ were a significant source of inspiration for him. He first came across this inscription while attending medical college, which has stayed with him as a lasting influence throughout his life [7]. Both his parents were ardent supporters of the Brahmo Samaj movement. He drew inspiration from this for the rest of his life. He began working for the State Health Service after completing his education. He aimed to pursue further education at St. Bartholomew's Hospital in London. Dr. Roy's application was denied, as he was an Indian. After 30 meetings with the institution's dean, it was eventually accepted. In two years and three months, Dr. Roy became the first person to finish the Fellowship of the Royal College of Surgeons (FRCS) in surgery and the Membership of the Royal Colleges of Physicians (MRCP) in medicine at St. Bartholomew's Hospital in London. He returned to India in 1911. In addition, Dr. Roy was a well-known liberation fighter and a close friend of Mahatma Gandhi. He was adamant that freedom would remain a pipe dream until people developed good physical and mental health.

Dr. Roy's political career

Dr. Roy began his political career in the elections of 1925. He worked for the Calcutta Corporation from 1930 to 1931 and was elected mayor in 1933. He built the city's infrastructure during this time and instituted free healthcare and education. Dr. Roy asserted that the youth of India would play a crucial role in determining the nation's future. He urged them to refrain from participating in strikes and fasts, advocating instead for a commitment to education and social work. In his convocation address at the University of Lucknow on 15 December 1956, he stated: ‘My young friends, you are soldiers in the struggle for freedom from want, fear, ignorance, frustration, and helplessness. Through dedicated efforts for the country, carried out in a spirit of selfless service, may you proceed with hope and courage’. Additionally, Dr. Roy strongly believed that Swaraj would remain a mere dream unless the citizens were healthy and strong in mind and body [8]. Upon the country's independence, he was appointed West Bengal's second chief minister in in 1948 and served in that role for 14 years until passing away. In addition, Dr. Roy is acknowledged as one of Bengal's modern architects. He focused on industrialising West Bengal, establishing several industries, including the Durgapur Steel Plant and the Kalyani engineering hub. He established numerous educational institutions, including the Indian Institute of Technology (IIT) Kharagpur, the first IIT in India. He worked to resettle millions of refugees who came to West Bengal following the partition of India. Dr. Roy promoted modern farming techniques and implemented land reforms to boost agricultural productivity. He founded industrial centres outside Calcutta and discovered five Bengali cities: Durgapur, Kalyani, Bidan Nagar, Ashok Nagar, and Habra. He provided stable governance during a tumultuous period, managing various political and social challenges. He was vital in establishing this multi-purpose river valley project for flood control, irrigation, and power generation. He initiated the planning and development of Salt Lake City (now Bidhannagar) as a planned satellite town of Kolkata.

A brilliant teacher and a scholar

In 1911, upon his return to India, he positioned himself as one of the most distinguished surgeons and doctors of his time. His exceptional medical skills and deep sense of humanity made him a respected and beloved figure among those seeking treatment or surgical care. Furthermore, he contributed to establishing various medical institutions throughout the nation, improving healthcare standards significantly. Dr. Roy transcended the role of a typical physician, distinguishing himself as both a proficient writer and a learned scholar. He wrote extensively on various medical topics, producing several research papers and multiple books intended for healthcare professionals. Additionally, his interests extended to psychology, the arts, and literature, reflecting the diverse capabilities of this talented Indian man [9]. In addition, he was a trailblazer in establishing the Medical Council of India (MCI) and the Indian Medical Association (IMA) in 1928. In addition, he was MCI's first president. As a result, he founded the Institute of Mental Health, the Infectious Disease Hospital, and the first postgraduate medical college in Calcutta.

Dr. Roy’s contributions to medical education and healthcare

Dr. Roy contested and won the Mayor of the Calcutta Municipal Corporation election. His administration was notable for its considerable impact on public health, highlighted by his leadership in the creation of several essential hospitals, such as Jadavpur TB Hospital, Kamala Nehru Memorial Hospital, and Chittaranjan Cancer Hospital, Victoria College, Chittaranjan Seva Sadan Hospital for women and children, along with a variety of smaller healthcare establishments in rural locales. His selection as the Vice-Chancellor of Calcutta University marked a significant period when he established several new colleges in the State, notably in the pharmacy field. In 1941, Dr. Roy was designated as the Governor of Bengal state. Despite his brief one-year term, he succeeded in introducing various improvements to the administrative structure [10]. He made significant efforts to elevate the standards of medical education at Kolkata Medical College, contributing to its development into one of the leading medical schools in the country. He played a crucial role in creating the IMA, which became a strong voice for the Indian medical community and promoted improved healthcare practices and policies. Dr. Roy was a founding member of the MCI, which established the nation's medical ethics and education guidelines. As he was interested in service towards cancer and women's and children's health, he established Chittaranjan Cancer Hospital, which later became one of India's leading cancer treatment centres, and Chittaranjan Seva Sadan Hospital for women and children. Dr. Roy was a key figure in founding Jadavpur University, which has grown into one of India's leading universities, mainly known for its engineering and technology programmes. During his tenure as chief minister, West Bengal's infrastructure development included the initiation and completion of several critical infrastructure projects, including the Durgapur industrial township and the expansion of Kolkata's urban facilities [11]. While Dr. Roy’s contributions to medicine were groundbreaking, his role in shaping West Bengal’s political landscape was equally transformative. His transition from healthcare to public service allowed him to implement policies extending his vision of a healthy, educated society.

Dr. Roy’s friendships and relationships

Dr. Roy maintained his single status, yet he was good friends with well-known people of his age. Dr. Roy first met Gandhi in 1917 and developed a deep, lasting friendship. Gandhi trusted Dr. Roy as both a friend and a physician. Dr. Roy often treated Gandhi during his fasts and provided him with medical advice. Their relationship was professional and ideological, as Dr. Roy was inspired by Gandhi's non-violence and social reform principles. His association with Mahatma Gandhi was especially noteworthy. He was respected and looked up to by Gandhi, who considered him to be his doctor. Gandhi and other leaders frequently sought Dr. Roy's medical guidance and personal counsel during pivotal stages in India's freedom struggle [12]. Dr. Roy and Nehru shared a warm personal friendship and a strong working relationship. They often corresponded on matters of national importance. Nehru respected Dr. Roy's expertise in development and healthcare, frequently consulting him on these issues. Their collaboration was crucial in shaping many post-independence policies.

Dr. Roy’s hobbies and interests

Dr. Roy, despite his demanding roles as a physician and politician, maintained a variety of hobbies and interests that reflected his multifaceted personality. An avid reader, he had a particular fondness for literature and philosophy, often spending his rare leisure moments immersed in books. Dr. Roy was also known to have a keen interest in gardening, finding solace in nurturing plants at his residence. Music was his passion, and he had a special appreciation for classical Indian compositions. He found comfort in music, especially Rabindranath Tagore's Rabindra Sangeet (songs). He enjoyed attending cultural events and encouraged the arts in West Bengal. Dr. Roy practised yoga regularly as a fitness enthusiast and believed strongly in maintaining a healthy lifestyle. He was also fascinated by architecture and urban planning, interests that influenced his vision for developing Kolkata and other parts of West Bengal. He was a philanthropist, deeply involved in various charitable activities, and was known for his simple lifestyle and dedication to public service. These diverse pursuits provided Dr. Roy with personal enjoyment and informed his holistic approach to governance and public service [10].

Awards and honours

Dr. Roy, a towering figure in Indian politics and medicine, received numerous prestigious awards and honours throughout his illustrious career, recognising his exceptional contributions to public service, medicine, and nation-building. The Government of India further honoured him with one of the highest civilian awards, the Padma Vibhushan, in 1955. The crowning glory of his accolades was the Bharat Ratna, India's highest civilian award, bestowed upon him posthumously in 1961, a testament to his unparalleled service to the nation [13]. In the field of medicine, Dr. Roy's expertise and dedication were acknowledged internationally when he was elected as a Fellow of the Royal College of Physicians of London and a Fellow of the American College of Chest Physicians, rare honours for an Indian doctor of his time. The British Medical Journal lauded him as the first medical consultant in the subcontinent of India, highlighting his pioneering role in the field. In recognition of his contributions to education, he was awarded the Doctor of Science (Honoris Causa) by the University of Calcutta. In 1962, Dr. Roy also received the prestigious Mountbatten Award from the Freedom from Hunger Campaign Committee, acknowledging his efforts in battling hunger and poverty. That same year, an annual prize named after Dr. Roy was established. The MCI established the National Award to recognize exceptional contributions to the fields of medicine, science, philosophy, politics, literature, and the arts [12]. To honour Dr. Roy, the MCI established the Dr. B.C. Roy National Award Fund in 1962. The Societies Registration Act of 1860 governs ‘the fund's’ registration. The Society's Memorandum of Association specifies the areas where ‘the fund’ accepts nominations. The award is worth Rs. 1,00,000 and comes with a silver salver in every category. His birthday, July 1, is celebrated as National Doctors' Day in India, an enduring tribute to his legacy in the medical profession. The Dr. B.C. Roy National Award was instituted in his memory by the MCI in 1976, annually recognizing excellent contributions to medicine, science, philosophy, literature, and politics. Additionally, numerous institutions, roads, and landmarks across India, particularly in West Bengal, have been named after him, serving as lasting memorials to his multifaceted contributions to Indian society. These honours not only reflect Dr. Roy's exceptional achievements but also underscore the profound impact he had on medicine, education, and public service in India [14].

Dr. Roy’s last days and legacy

In his final days, Dr. Roy exhibited remarkable grace and resilience, reflecting on the journey that shaped his career and the lives he had transformed. Surrounded by family, friends, and colleagues, he shared stories of the challenges he faced and the breakthroughs he achieved, emphasizing the importance of empathy in medicine. As he prepared to leave this world, he expressed a deep sense of fulfilment, knowing that his pioneering research had paved the way for new treatments and improved patient outcomes. Dr. Roy died on 1 July 1962, a date that coincidentally marked his birth. After his death, his home was converted into a nursing home named after his mother, Aghore Kamini Devi. He also established a trust for his properties in Patna to further social service activities [15]. His legacy extends beyond his scientific contributions; it lives on in the countless medical professionals he mentored, inspiring them to prioritise holistic care and community engagement. Dr. Roy’s commitment to his patients and the ideals he championed will continue to resonate in the medical field, serving as a beacon of compassion and innovation for years to come.

## Conclusions

Dr. Roy was a multifaceted individual recognised for his work as a physician, educator, teacher, humanist, administrator, and politician. He was a prominent figure in India's history and made significant contributions as a physician, freedom fighter, and statesman. As one of the most esteemed doctors of his time, he was instrumental in advancing medical education and healthcare in India, founding institutions such as the IMA and the MCI. His role as the second chief minister of West Bengal was marked by his visionary leadership, where he focused on rebuilding the state post-independence, promoting industrialisation, and improving infrastructure. Dr. Roy’s contributions to healthcare and politics remain foundational to modern India. His work established medical institutions and infrastructure, improved healthcare access, and laid the groundwork for public health policy in the post-colonial era. His legacy continues to inspire medical professionals and leaders alike.

## References

[1] (2024). Back to the beginning - on the 50th year of the landfill, here’s the story of how Salt Lake came into being. https://www.telegraphindia.com/west-bengal/back-to-the-beginning-on-the-50th-year-of-the-landfill-heres-the-story-of-how-salt-lake-came-into-being/cid/396434.

[2] (2024). Who was Dr B.C. Roy? Here's why National Doctors Day honours West Bengal's first chief minister. https://www.theweek.in/news/health/2024/07/01/who-was-dr-bc-roy-heres-why-national-doctors-day-honours-west-bengals-first-chief-minister.html.

[3] (2024). National Doctor’s Day special- celebrating Dr Bidhan Chandra Roy: least explored journalist of India. https://medicaldialogues.in/articles/national-doctors-day-special-celebrating-dr-bidhan-chandra-roy-least-explored-journalist-of-india-130956.

[4] (2024). Dr. Bidhan Chandra Roy: the reason India celebrates Doctors’ Day. https://www.newsbytesapp.com/news/lifestyle/doctor-s-day-remembering-dr-bidhan-chandra-roy-s-legacy/story.

[5] (2024). File:Photograph of Dr. Bidhan Chandra Roy, 2nd Chief Minister of West Bengal.jpg - Wikimedia Commons. https://commons.wikimedia.org/wiki/File:Photograph_of_Dr._Bidhan_Chandra_Roy,_2nd_Chief_Minister_of_West_Bengal.jpg.

[6] (2024). 25 fascinating facts about Dr Bidhan Chandra Roy - the checkup. https://www.thecheckup.in/25-fascinating-facts-about-dr-bidhan-chandra-roy.

[7] Bureau EN. Dr Bidhan Chandra Roy (2024). Dr Bidhan Chandra Roy - a leader par excellence express healthcare. Express Healthcare.

[8] (2024). Tribute to legendary Dr BC Roy. https://opinionexpress.in/tribute-to-legendary-dr-bc-roy.

[9] Dr Bidhan Chandra Roy (2024). Dr Bidhan Chandra Roy biography. https://www.quotagebiography.com/dr-bidhan-chandra-roy-1882-1962/.

[10] Dr. B.V. Rajagopal (2024). National Doctors Day today: a tribute to Bharat Ratna Dr. Bidhan Chandra Roy. Star of Mysore.

[11] (2024). Who was Bidhan Chandra Roy? Everything you need to know. https://www.thefamouspeople.com/profiles/bidhan-chandra-roy-7454.php.

[12] (2024). Bidhan Chandra Roy: remembering the man behind National Doctor's Day. News9live [Internet.

[13] (2024). History today in medicine-Dr. Bidhan Chandra Roy. https://cmeindia.in/history-today-in-medicine-dr-bidhan-chandra-roy/.

[14] Dr. B.C (2024). Dr. B.C Roy Award | NMC. https://www.nmc.org.in/awards/dr-b-c-roy-award/.

[15] Prasad R (2024). Speeches of president Rajendra Prasad. Central Archeological library.

